# Lemierre’s Syndrome in the External Jugular Vein Precipitated by COVID-19 Infection

**DOI:** 10.7759/cureus.34512

**Published:** 2023-02-01

**Authors:** Raghavendra R Sanivarapu, Ramya Sruthi Rajamreddy, Swetha Nalla, Shameera Shaik Masthan, Goutami Mangu

**Affiliations:** 1 Pulmonary and Critical Care Medicine, Texas Tech University Health Sciences Center, Odessa, USA; 2 Pulmonary and Critical Care Medicine, Nassau University Medical Center, East Meadow, USA; 3 Internal Medicine, People's Education Society Institute of Medical Sciences and Research (PESIMSR), Kuppam, IND; 4 Internal Medicine, Mamata Medical College, Khammam, IND; 5 Medicine, University of Louisville, Kentucky, USA; 6 Internal Medicine, Texas Tech University Health Sciences Center, Odessa, USA

**Keywords:** lemierre’s syndrome, sars-cov-2 (severe acute respiratory syndrome coronavirus-2), external jugular vein thrombosis, coronavirus disease 2019 (covid-19), jugular vein thrombophlebitis

## Abstract

Lemierre’s syndrome is a condition when an oropharyngeal infection, typically from *Fusobacterium necrophorum*,* *causes thrombophlebitis of the internal jugular vein. There have been few case reports of Lemierre's syndrome affecting the external jugular vein, but to our knowledge, this is the first case report where COVID-19 infection is the prime suspect for causing this syndrome. SARS-CoV-2 infection, known to cause hypercoagulability and immunosuppression, increases the risk of deep venous thrombosis and secondary infections. We report a case of a young male with no known risk factors who developed Lemierre’s syndrome as a complication of COVID infection.

## Introduction

First described by Dr. Andrè Lemierre in 1936, Lemierre’s syndrome is an oropharyngeal infection with septic thrombophlebitis of the internal jugular vein (IJV) caused mainly by *Fusobacterium necrophorum*, an anaerobic gram-negative rod [1]. There have been multiple case studies of Lemierre’s syndrome affecting the external jugular vein (EJV) caused by alpha-hemolytic streptococcus [1-3], but to the best of our knowledge, this is the first case report of Lemierre’s syndrome due to COVID-19 infection. In this article, we report a case of a young male with no known risk factors and a recent COVID-19 infection diagnosed with Lemierre’s syndrome of the EJV.

## Case presentation

A 31-year-old male with no significant medical history and no intravenous (IV) drug abuse was transferred to our facility for evaluation of left-sided neck pain and swelling. The patient had a fever and upper respiratory infection (URI) a week ago before developing neck pain and swelling. At admission, his vitals were temp 100.4°F, heart rate 116 bpm, blood pressure 120/81 mm Hg, and SpO_2_ 94% on room air. His physical exam was only significant for tenderness of the left side of the neck and engorged veins with swelling.

His initial blood work was significant for leukocytosis 17.6 x 10^3^/μL with neutrophilia at 76% and lymphopenia at 14.7%, and the C-reactive protein was 16.3 mg/dL. Another significant finding was that his A1c was > 14.0%. A drug screen was negative, and other labs including electrolyte panels and cultures were unimpressive.

**Figure 1:**
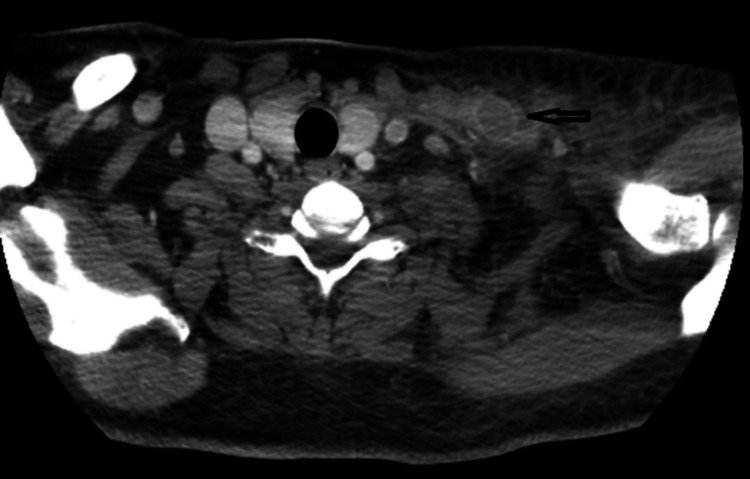
CT scan of neck axial view showing thrombus in the left external jugular vein (black arrow)

**Figure 2:**
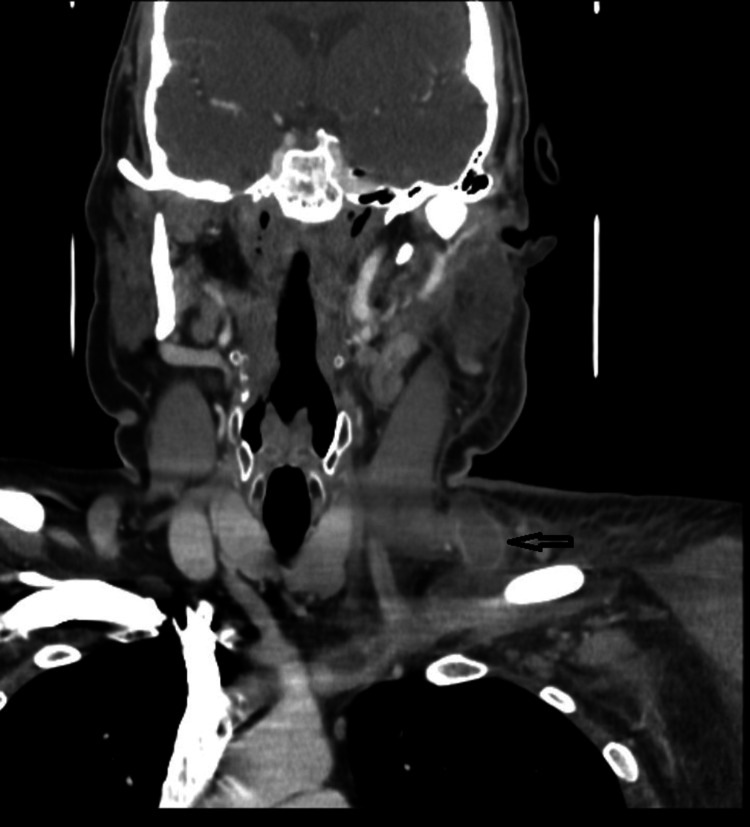
CT scan of the neck coronal section showing thrombus in the left external jugular vein and swelling of left side soft tissue

Computed tomography (CT) of the neck confirmed an occluded left EJV with a thrombus (Figures 1, 2) extending into the left subclavian vein, brachiocephalic vein, and the superior mediastinum with patchy bilateral airspace opacities consistent with multilobar pneumonitis.

**Figure 3:**
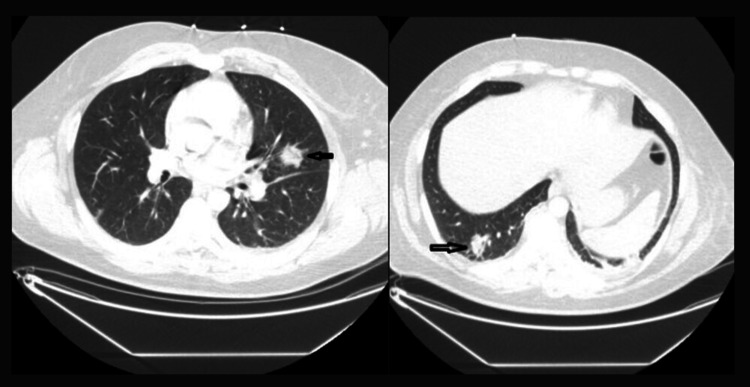
CT thorax showing septic emboli bilaterally (arrows)

CT angiography of the chest showed the presence of bilateral septic emboli (Figure 3). A duplex ultrasound confirmed an acute thrombus of the left subclavian vein. A 2D echocardiogram showed an ejection fraction of >60%, with no signs of endocarditis.

The patient was started on treatment for septic thrombophlebitis. His SARS-CoV-2 polymerase chain reaction study was negative, but antibodies were positive for IgG with quantitative IgG of 58.0 BAU/mL, establishing recent COVID-19 infection, given his upper respiratory infection (URI) symptoms a week prior.

All his cultures remained negative, likely because of the early initiation of antibiotics before the samples were drawn. He was initially treated with linezolid, piperacillin + tazobactam, and clindamycin with no significant improvement.

His hospital stay was complicated with parotitis and a lack of clinical improvement, necessitating escalation of antibiotics to meropenem and continuation on linezolid. Then, the patient showed significant improvement in clinical status with a reduction in his parotid swelling and induration on the left face and neck. We discharged the patient on apixaban and ertapenem for four weeks, with a follow-up appointment.

## Discussion

Lemierre's syndrome refers to inflammation of the wall of the IJV and infected thrombus in the lumen caused by an oropharyngeal infection with surrounding soft tissue swelling and inflammation [4,5]. The incidence of the disease is found to be 14.4 cases per million people among the age groups of 14 to 24 years [6]. A few case reports have reported inflammation of the EJV with thrombosis [1-3].

The common organism to cause Lemierre’s syndrome is *Fusobacterium necrophorum*, a nonmotile, filamentous, non-spore-forming gram-negative bacillus [7]. Other organisms that are known to cause Lemierre’s syndrome include *Eikenella corrodens*, *Porphyromonas asaccharolytica*,and *Bacteroides* [8-10]. The proposed mechanism by which oropharyngeal organisms cause septic thrombophlebitis is postulated to be via hematogenous spread through a tonsillar vein or lymphatics. The involvement of EJV thrombophlebitis has been reported to be caused by alpha-hemolytic streptococci in multiple case reports. Our case presents a unique scenario of recent COVID-19 infection directly causing or provoking septic thrombophlebitis uniquely in the EJV. Infection with SARS-CoV-2 is known to cause a prothrombotic state from endothelial injury and changes in circulating prothrombotic factors like elevated factor VIII, fibrinogen, neutrophil extracellular traps, and hyperviscosity.

Common clinical manifestation includes fever, sore throat, dysphagia, unilateral neck pain, and tenderness. Most patients have prior parotitis or an upper airway infection. Our patient presented with left-sided neck pain, swelling, and tenderness that started after an upper respiratory infection a week prior, which is presumed to be a COVID-19 infection due to his high antibody titers. The thrombus can embolize, cause septic emboli in the lungs, and present as dyspnea, pleurisy, or sometimes with hemoptysis [11]. Our patient’s CT thorax showed the presence of septic emboli, which is likely from embolization.

The diagnosis is established in the presence of radiographic imaging demonstrating thrombus in neck veins, usually IJV and positive culture results. CT angiography of the neck and chest will provide diagnostic evaluation and can identify thrombosis and the presence of septic emboli in the lungs [12].

The treatment includes initiation of appropriate antibiotics and anticoagulation, and surgical intervention may be considered in some cases. The usual antibiotic regimen must include piperacillin-tazobactam as *Fusobacterium *is a beta-lactamase-producing organism. In cases that do not respond, antibiotics can be escalated to carbapenems such as imipenem, which was the case in our patient [13,14]. The duration of antibiotics depends on clinical recovery and is usually continued for at least four weeks during which intravenous antibiotics are given for two weeks. The data on the use of anticoagulation is limited as it is not known to reduce thrombus propagation or septic emboli and is usually reserved for patients with severe infection and propagating thrombus, as in our case [15,16].

## Conclusions

Typical Lemierre's syndrome usually involves IJV, and EJV involvement is rare with only a few cases reported in the literature. The patient likely had a recent COVID-19 infection with negative PCR and positive antibodies. This forgotten and life-threatening condition was likely triggered in this patient by a recent SARS-CoV-2 infection and uncontrolled diabetes. Prompt diagnosis and initiation of appropriate antibiotics remain the mainstay of treatment. Physicians should be very diligent about when to use anticoagulation, especially in cases with progressive thrombosis and septic emboli. Lemierre's syndrome caused by bacteria is well-known and has been reported in many cases, but to our best knowledge, this is the first case of COVID-19-precipitated Lemierre's syndrome that also presented in the EJV.
